# Exercise and Atrial Fibrillation: Current Evidence, Knowledge Gaps, and Future Directions

**DOI:** 10.31083/RCM39200

**Published:** 2025-07-28

**Authors:** Hoang Nhat Pham, Mahmoud H. Abdelnabi, Ramzi Ibrahim, Enkhtsogt Sainbayar, Hong Hieu Truong, Eiad Habib, Girish Pathangey, George Bcharah, Amitoj Singh, Reza Arsanjani, Anwar A. Chahal, Dan Sorajja

**Affiliations:** ^1^Department of Medicine, University of Arizona, Tucson, AZ 85721, USA; ^2^Department of Cardiovascular Medicine, Mayo Clinic, Rochester, MN 55901, USA; ^3^Department of Cardiovascular Medicine, Mayo Clinic, Phoenix, AZ 85054, USA; ^4^Department of Internal Medicine, Ascension Saint Francis, Evanston, IL 60202, USA; ^5^Mayo Clinic Alix School of Medicine, Phoenix, AZ 85054, USA; ^6^Department of Cardiology, Sarver Heart Center, University of Arizona, Tucson, AZ 85721, USA; ^7^Center for Inherited Cardiovascular Diseases, WellSpan Health, York, PA 17403, USA; ^8^Barts Heart Centre, EC1A 7BE London, UK; ^9^William Harvey Research Institute, Queen Mary University of London, E1 4NS London, UK

**Keywords:** atrial fibrillation (AF), exercise intensity, moderate-intensity exercise, endurance exercise, AF burden, cardiorespiratory fitness (CRF), cardiovascular risk

## Abstract

Atrial fibrillation (AF) is the most common sustained cardiac arrhythmia, contributing to significant morbidity and mortality worldwide. The relationship between physical exercise and AF is complex, with studies showing both beneficial and potentially adverse effects. Moreover, evidence suggests a U-shaped association between exercise intensity and AF risk. Moderate exercise has been shown to reduce AF burden by improving cardiovascular risk factors, enhancing autonomic regulation, and mitigating atrial fibrosis. In contrast, excessively high-intensity endurance exercise may increase AF risk, particularly in young athletes, due to atrial stretching, dilation, fibrosis, autonomic imbalances, and heightened inflammation. The current guidelines emphasize exercise as a core lifestyle intervention for AF management, recommending moderate-intensity aerobic activity for optimal outcomes. This review examines the current evidence on the effects of exercise on AF, identifies knowledge gaps, and proposes potential future research directions.

## 1. Introduction

Atrial fibrillation (AF) is the most common, sustained cardiac arrhythmia, contributing to a significant and growing global health burden [1]. In 2020, the estimated global prevalence of AF was approximately 50 million and is projected to increase further. This surging burden is driven by multiple factors, including the aging population, rising obesity rates, improved detection methods, and enhanced survival among individuals with AF and other cardiovascular diseases [2, 3]. AF is associated with increased morbidity and mortality, increasing the risk of stroke by 2.4-fold, heart failure by 5-fold, and overall mortality by 1.5- to 2-fold [1]. Prior literature has shown that physical exercise improves clinical outcomes in several cardiovascular diseases by reducing mortality and hospitalizations while enhancing cardiac function and quality of life across various exercise types and patient populations [4, 5, 6]. However, the relationship between exercise and AF is complex. It has a dose-dependent effect, with both beneficial and potentially adverse effects [7]. Evidence suggests a U-shaped relationship between exercise intensity and AF risk, with moderate exercise being protective and high-intensity endurance exercise potentially increasing the risk, particularly in athletes [8]. This review aimed to discuss the current understanding of the impact of different exercise intensities and modalities on AF and identify evidence gaps and future research directions.

## 2. Definition and Characteristics of Exercise

The 2020 European Society of Cardiology (ESC) Guidelines on Sports Cardiology and Exercise define exercise as a subset of physical activity that is planned, structured, repetitive, and purposeful, to improve or maintain physical fitness [9]. The fundamental principles of exercise prescription follow the “FITT” concept (frequency, intensity, time, and type), with the mode of exercise also being a key characteristic [9]. Exercise types are traditionally categorized into endurance and resistance training, which can be further classified based on modes of exercise, including metabolism (aerobic or anaerobic) and muscle contraction types (isotonic-isometric, dynamic-static, continuous-interval, or large-small muscle groups) [9]. Aerobic exercises, such as cycling, running, or swimming at low or moderate intensity, involve large muscle groups performing dynamic activities, significantly increasing heart rate and energy expenditure. These activities primarily rely on oxygen-dependent energy pathways [10, 11]. Aerobic training can be continuous or interval-based, with interval training offering greater physiological benefits but requiring careful application in cardiac patients due to the increased stress on the cardiovascular system [12]. In contrast, anaerobic activities, such as intermittent high-intensity exercise, require sustained isometric muscle and rely on anaerobic glycolysis for energy production, rather than oxygen [10, 11]. Exercise frequency is measured in sessions per week, with guidelines recommending moderate exercise on most days, totaling at least 150 minutes per week [9]. Exercise intensity is the most critical for achieving aerobic fitness and for having the most favorable impact on risk factors [13]. Exercise Intensity can be measured by energy expenditure (metabolic equivalents [METs]), percentage of maximal aerobic capacity (maximum oxygen consumption [VO_2_ max]), heart rate (percentage of maximum heart rate [HRmax] or percentage of heart rate reserve [HRR]), rate of perceived exertion (Borg scale) or “talk test” (Table 1, Ref. [9, 10, 11, 14]). Training volume is determined by the product of intensity and duration, with gradual progression recommended (increase weekly either by 2.5% in intensity or 2 minutes duration) [9, 15].

**Table 1.** **Classification of exercise intensity based on physiological and perceived exertion metrics [9, 10, 11, 14]**.

Intensity	METs	VO_2_ max	HRmax (%)	HRR (%)	RPE scale
Low/Light intensity	1.6–2.9	<40	<55	<40	10–11
Moderate intensity	3.0–5.9	40–69	55–74	40–69	12–13
High intensity	≥6	70–85	75–90	70–85	14–16
Very high intensity		>85	>90	>85	17–19

Abbreviation: HRmax, maximum heart rate; HRR, heart rate reserve; METs, metabolic equivalents; RPE, rate of perceived exertion; VO_2_ max, maximum oxygen consumption.

## 3. Benefits of Exercise in AF Management

The benefits of exercise for AF are multifaceted and attributable to several mechanisms, primarily through lowering cardiovascular risk factors and modifying atrial substrate. Regular moderate exercise lowers heart rate, blood pressure, and improves glucose and lipid control, thereby reducing overall cardiovascular risk, a major contributor to AF development [8]. Additionally, weight management via exercise enhances cardiorespiratory fitness (CRF), which has been associated with a significant reduction in AF burden and symptom severity, particularly in patients with obesity [16]. Beyond metabolic benefits, exercise exerts anti-inflammatory effects through pathways including cytokine modulation, AMP-activated protein kinase activation, and autonomic regulation; collectively reducing systemic inflammation, a known driver of AF pathogenesis [17, 18, 19]. Endothelial dysfunction plays a critical role in the pathophysiology of AF by promoting an atrial arrhythmic substrate and predicting adverse outcomes [20, 21, 22]. Exercise enhances endothelial function, which supports vascular health and reduces atrial stretch and fibrosis, thereby mitigating AF progression [8]. Prior studies in both rats and humans indicated that aerobic interval training can decrease age-related atrial fibrosis, a significant factor in AF vulnerability [23, 24]. This reduction in fibrosis leads to improved atrial conduction velocity and longer action potentials, ultimately decreasing susceptibility to AF [23]. Furthermore, regular exercise can improve autonomic balance by increasing parasympathetic activity and reducing sympathetic activity, stabilizing the atrial electrical environment [8]. Animal studies have demonstrated that exercise training restores parasympathetic modulation, improves baroreceptor reflex control, and enhances cardiac vagal tone, even with persistent ventricular dysfunction [25, 26].

The 2023 American College of Cardiology (ACC)/American Heart Association (AHA)/American College of Chest Physicians (ACCP)/Heart Rhythm Society (HRS) guidelines introduce a paradigm shift in AF management, emphasizing personalized risk factor modification and lifestyle interventions, with physical inactivity identified as one of the key targets [1, 27]. Regular physical exercise offers numerous benefits for patients with AF, including reducing arrhythmia burden, improving symptoms, lowering AF recurrence, improving functional capacity and quality of life (QoL), and lowering mortality and morbidity risks [1, 27]. These benefits are supported by several randomized clinical trials (RCTs). The CARDIO-FIT study enrolled 308 obese individuals with AF, with a mean age of 61 ± 9 years, and a mean body mass index (BMI) of 33 ± 5 kg/m^2^, demonstrating that improved CRF significantly reduces arrhythmia recurrence. The greatest arrhythmia-free survival was observed in those who achieved a fitness gain of ≥2 METs, as those patients had an 89% arrhythmia-free survival rate compared to 40% for those patients who gained <2 METs (*p* < 0.001). In fact, every MET gained was associated with a 9% decline in the risk of arrhythmia recurrence even after multifactorial adjustment (hazard ratio [HR] per 1-MET change: 0.90; 95% CI, 0.83–1.00; *p* = 0.036). Additionally, AF burden and symptom severity were notably lower in patients with high CRF compared to those with adequate or low fitness levels [28]. Prior studies have also shown that moderate-intensity aerobic training reduced AF incidence and burden in patients with non-permanent (paroxysmal and persistent) AF, and improved exercise capacity and overall QoL in those with non-permanent and permanent AF [1, 29]. Similarly, aerobic continuous training (moderate-intensity continuous training, MICT) has been linked to a lower risk of developing AF, with HRs ranging from 0.72 to 0.90 compared to physical inactivity. MICT has also been shown to reduce symptom burden and enhance QoL [1, 30, 31]. High-intensity interval training (HIIT), alternating short bursts of high-intensity exercise with recovery periods, offers comparable or slightly better improvements in CRF and symptom control than MICT. However, its long-term safety in AF is less established, and even with some evidence of elevated AF occurrence in patients with hypertension or kidney disease [30, 32]. Though not extensively studied, resistance training, especially moderate-intensity isometric and isotonic exercises, is generally safe for most AF patients without structural heart disease and can be part of a broader exercise regimen. However, its direct effect on AF burden remains unclear [33].

The ACTIVE-AF (A Lifestyle-based, PhysiCal AcTIVity IntErvention for Patients With Symptomatic Atrial Fibrillation) RCT, which followed 120 patients with paroxysmal or persistent (non-permanent) AF over 12 months, demonstrated that a 6-month exercise-based intervention combining home and supervised aerobic exercise reduced AF recurrence in both men (HR 0.52 [95% CI, 0.29–0.91]) and women (HR 0.47 [95% CI, 0.23–0.95]). Furthermore, symptom severity scores were significantly lower in the exercise group compared to controls, with women experiencing the highest benefit [16]. Moreover, a nationwide population-based cohort study in Korea showed that patients with newly diagnosed AF who started or maintained regular exercise had significantly lower risks of heart failure (HR 0.95 [95% CI, 0.90–0.99] and 0.92 [95% CI, 0.88–0.96], respectively) and all-cause mortality (HR 0.82 [95% CI, 0.73–0.91] and 0.61 [95% CI, 0.55–0.67], respectively) compared to non-exercisers [34]. Several meta-analyses have further reinforced these findings. A meta-analysis from thirteen RCTs by Oesterle *et al*. [35] found that supervised exercise training significantly reduces AF recurrence (relative risk 0.77 [95% CI, 0.60–0.99]) and improves QoL and CRF in participants with AF. This analysis included various exercise modalities, including cardiac rehabilitation, aerobic training, and yoga, highlighting the broad applicability of exercise-based interventions [35]. Another meta-analysis by Shi *et al*. [36] further highlighted improvements in physical functioning (standardized mean difference [SMD] 0.63 [95% CI, 0.18–1.09]; *p* = 0.006), general health perceptions (SMD 0.64 [95% CI, 0.35–0.93]; *p* < 0.001), vitality (SMD 0.51 [95% CI, 0.31–0.71]; *p* < 0.001), as well as increased performance in the 6-minute walk test (SMD 0.69 [95% CI, 0.19–1.11]; *p* = 0.007) and peak oxygen uptake (SMD 0.37 [95% CI, 0.16–0.57]; *p* < 0.001). Much of the benefit may be derived from the extended duration of physical exercise, being at least 60 minutes per session [36]. These findings highlight the integral role of exercise in AF management and provide strong evidence for its implementation as a core therapeutic strategy.

## 4. Risks Associated With High-Intensity Exercise

The increased risk of AF associated with high-intensity, long-duration exercise is not fully understood. This phenomenon involves a complex interplay of atrial structural and electrical changes, increased vagal tone, and heightened inflammation [37]. Chronic endurance exercise leads to atrial dilation and fibrosis, driven by elevated atrial pressures and stretch from repeated strenuous activity [38, 39]. This mechanical stress causes microtears and subsequent fibrosis, contributing to atrial remodeling and creating a substrate conducive to AF [40]. Experimental models have shown atrial-specific extracellular matrix remodeling in endurance exercise, with increased collagen deposition and fibrosis, particularly in the right atrium. This process is load-dependent and correlates with increased AF susceptibility [39, 41]. Molecular studies in animal models have also shown the upregulation of transforming growth factor-beta 1 (TGF-β1) and matrix metalloproteinase 2 (MMP-2) in both atria after 16 weeks of intensive exercise, paralleling increased collagen content and fibrosis [39, 42]. In 1 human study, serum TGF-β levels have been found to correlate with left atrial volume and reduced strain in athletes, supporting its role as a biomarker of subclinical fibrosis [43]. Excessive endurance exercise may also increase vagal tone to a level that promotes AF through vagal-mediated remodeling [41, 44]. This autonomic imbalance prolongs atrial conduction while shortening the atrial refractory period, further predisposing to AF [39]. Inflammatory mechanisms also play a role, as prolonged endurance exercise has been associated with elevated levels of C-reactive protein and troponin, indicating systemic inflammation [45]. This inflammatory response, mediated through TNFα-dependent pathways involving NFκB and p38MAPK activation, promotes atrial hypertrophy, fibrosis, and macrophage infiltration, creating a pro-arrhythmic substrate for AF [44, 46, 47]. Furthermore, endurance exercise affects ion channel expression and function (ion channel remodeling), with downregulation of L-type calcium channels (ICaL) and an increase in inward rectifier potassium current (IK1), leading to shortened action potentials and increased susceptibility to reentry [39]. Enhanced sensitivity of the acetylcholine-regulated potassium current (IKACh) further increases vagal tone, increasing predisposition to AF [39].

Several studies have suggested that while moderate exercise is protective against AF, excessive endurance training, particularly among elite athletes and individuals engaging in long-term, high-intensity exercise, may paradoxically increase AF risk [41, 48]. The 2023 ACC/AHA/ACCP/HRS guidelines note that high-volume endurance athleticism (defined as greater than 45 metabolic equivalent hours per week) is associated with a higher prevalence of AF, particularly in young athletes [1]. Epidemiological data indicate a significantly higher prevalence of AF among endurance athletes, such as marathon runners and cyclists, compared to the general population, with an odds ratio (OR) of 2.34. This risk is more pronounced in men (OR 4.03 [95% CI, 1.73–9.42]) and younger athletes under 60 years (OR 3.24 [95% CI, 1.23–8.55]) [49]. However, elite female endurance athletes also face an elevated risk compared to the general population, with an adjusted HR of 3.67 (95% CI, 1.71–7.87), highlighting that this association between intense exercise and AF extends to both sexes [50]. A 12-week RCT in 76 patients with paroxysmal/persistent AF found that high-intensity physical exercise was not superior to low-intensity physical exercise in reducing the burden of AF (incidence rate ratio 0.742 [95% CI, 0.29–1.91]) [51]. Furthermore, extreme levels of exercise have been associated with a higher incidence of AF. An observational cohort study of 52,755 long-distance cross-country skiers with a 16-year follow-up (1989–2005) found a higher AF risk among male participants with greater race participation (HR 1.29 [95% CI, 1.04–1.61]) and faster finishing times (HR 1.20 [95% CI, 0.93–1.55]) [52].

This relationship between AF and high-intensity exercise appears to be dose-dependent, following a U-shaped pattern, with the highest incidence observed in individuals with extensive training histories or significant cumulative lifelong exercise exposure [1]. Specifically, those with a history of 2000 or more hours of cumulative high-intensity endurance training have a higher risk of AF compared to sedentary individuals (OR 3.88 [95% CI, 1.55–9.73]) [53]. However, HIIT may improve functional capacity and QoL without increasing AF burden compared to moderate to vigorous intensity continuous training [32]. Interpretation of these findings should consider the heterogeneity in methods used to assess exercise intensity, including self-reported data, which is inherently prone to recall bias.

Anticoagulation management in patients with AF who participate in high-intensity exercise also presents a unique challenge, requiring balancing the prevention of thromboembolic events with the risk of bleeding, particularly from exercise-related injuries [1, 54, 55]. Previous studies have shown that the CHA_2_DS_2_-VASc score may be a useful tool for assessing exercise intolerance in patients with AF. Those with exercise intolerance had significantly higher scores compared to those without (3.1 ± 1.3 vs. 2.0 ± 1.5, *p* < 0.0001), with the effect being more pronounced in younger and middle-aged male patients under 65 years old (OR 3.30, 95% CI, 1.76–6.19, *p* < 0.0001) [56]. Anticoagulation decisions should be individualized based on the specific sport, trauma risk, and the patient’s preferences. Direct oral anticoagulants (DOACs) are generally preferred over vitamin K antagonists due to their more predictable effects and lower risk of bleeding complications [57]. It is important to consider that pharmacologic rate control agents, such as beta-blockers, may reduce exercise tolerance and are less favored in this population due to their potential to hinder athletic performance [58]. In contrast, rhythm control strategies, including catheter ablation, are often prioritized to maintain exercise capacity and improve quality of life. This approach is especially beneficial for young athletes who may not tolerate or prefer to avoid long-term medication use [1].

## 5. Sex Difference in AF Risk With Exercise

Evidence suggests that high levels of vigorous physical activity in men are associated with an increased risk of developing AF. A meta-analysis supported this finding, showing that men engaging in vigorous physical activity had a significantly increased risk of AF (pooled OR 3.30, 95% CI, 1.97–4.63) [59]. Another study concluded that male athletes have more pronounced atrial remodeling and higher sympathetic tone, possibly contributing to the increased risk of AF [60]. Conversely, moderate to intense physical activity in women can be protective against AF. Women performing moderate physical activity were found to have an 8.6% lower risk of developing AF compared to those with a sedentary lifestyle (OR 0.91, 95% CI, 0.78–0.97), and those engaging in intense exercise had a 28% lower risk (OR 0.72, 95% CI, 0.57–0.88) [59]. Additionally, a meta-analysis found that total physical activity exposure was associated with a decreased risk of AF in women (relative risk 0.92, 95% CI, 0.87–0.97) [61]. These differences could be attributable to sex differences in autonomic adaptations to exercise, particularly increased parasympathetic tone, with men having more pronounced autonomic changes and being more susceptible to AF triggered by high vagal tone from endurance exercise, while women appear less prone to this effect [62, 63]. Other contributing factors may include sex-related differences in exercise-induced hormonal modulation or inflammatory responses, though these mechanisms are not well characterized in clinical studies and warrant further investigation [45, 64]. These age and gender-specific differences highlight the importance of tailored exercise recommendations based on age and sex to reduce the risk of AF.

## 6. Guidelines and Recommendations

The 2023 ACC/AHA/ACCP/HRS guidelines recommend moderate- to vigorous-intensity exercise, with a target of 210 minutes per week for individuals with AF. Aerobic activities such as walking, cycling, or swimming are preferred and can be divided into sessions of 30 minutes on most days of the week. This exercise regimen aims to reduce the frequency and duration of AF episodes while improving cardiorespiratory fitness and symptom severity [1, 16]. Before prescribing exercise, an initial evaluation should be conducted to determine the patient’s exercise capacity and identify any comorbid conditions that may affect exercise tolerance [1]. Exercise prescription should be tailored based on patients’ comorbidities, such as obesity, hypertension, and diabetes, to optimize outcomes [28]. For example, AF patients with obesity should focus on achieving significant weight loss through a combination of aerobic and resistance training, with a target of at least 10% weight loss to reduce AF symptoms, burden, recurrence, and progression to persistent AF [1, 28, 65, 66]. For AF patients with diabetes, a gradual approach is recommended, starting with low-to-moderate-intensity activities such as walking, cycling, or swimming and gradually increasing in duration and intensity as tolerated [1]. Furthermore, exercise training may also be incorporated into multidisciplinary cardiac rehabilitation, which has been shown to improve QoL and functional capacity without increasing the risk of AF recurrence, particularly in patients undergoing ablation [67, 68]. HIIT may also be considered in selected patients, provided it is well-tolerated and monitored closely [7]. In patients with hypertension or chronic kidney disease, moderate-intensity exercise is preferred, since HIIT may paradoxically increase AF occurrence in these subgroups [30].

Regular monitoring of cardiovascular status is essential, including heart rate or symptoms such as palpitations or dizziness, as well as blood glucose levels, especially in patients with diabetes. The American Heart Association recommends targeting a heart rate of <80 bpm at rest and <110 bpm during moderate exercise to improve ventricular function and exercise capacity and to prevent tachycardiomyopathy [69]. Careful ventricular rate control is necessary during exercise to prevent atrial myopathy associated with excessive training [1]. Patients should be educated on recognizing symptoms that warrant cessation of exercise and medical consultation. High-intensity activities that may exacerbate AF or cause significant blood glucose fluctuations should be avoided [1, 70]. The exercise regimen should be adjusted based on the patient’s response during follow-up visits, particularly in those with heart failure or coronary artery disease [1].

The 2020 ESC Guidelines on sports cardiology and exercise recommend engaging in regular physical activity, with at least 150 minutes of moderate-intensity aerobic exercise per week, to prevent AF [9]. While both the 2023 ACC/AHA and 2020 ESC guidelines emphasize the benefits of moderate-intensity exercise in reducing AF burden and improving cardiovascular fitness, the 2020 ESC explicitly cautions against excessive high-intensity endurance training, especially for middle-aged men, due to its potential to increase AF risk [8, 9]. Additionally, the 2020 ESC guidelines highlight the importance of evaluation and management of underlying conditions, such as structural heart disease, thyroid dysfunction, alcohol or drug abuse, or other primary causes of AF, before engaging in sports [9]. In contrast, the 2023 ACC/AHA guidelines provide more detailed recommendations for tailoring exercise regimens based on comorbidities, such as obesity and diabetes [1]. Both guidelines emphasize the importance of rate control during exercise, but the 2023 ACC/AHA guidelines specify target heart rate thresholds to ensure safety and performance optimization [1, 9]. Finally, the ESC also provides specific recommendations on sports participation for anticoagulated patients, advising against high-contact or trauma-prone activities [9]. The 2020 Canadian Cardiovascular Society guidelines recommend that AF patients engage in at least 200 minutes per week of moderate-intensity aerobic exercise, complemented by resistance and flexibility training. These guidelines emphasize the importance of individualized exercise prescriptions, considering factors such as exercise intensity, duration, and patient-specific characteristics [71]. Guidelines recommendations are summarized in Table 2.

**Table 2.** **Exercise recommendations for atrial fibrillation management: guidelines overview**.

Name of guideline	Recommendations
2023 American College of Cardiology, American Heart Association, American College of Chest Physicians, Heart Rhythm Society	Engage in moderate-to-vigorous exercise training for a target of 210 minutes per week to reduce AF symptoms, enhance sinus rhythm maintenance, improve functional capacity, and boost quality of life.
2020 ESC Guidelines on sports cardiology and exercise in patients with cardiovascular disease	At least 150 minutes of moderate-intensity aerobic activity is recommended to prevent atrial fibrillation (AF), with caution against excessive high-intensity endurance training due to a potential increased risk of AF, especially in middle-aged men. Evaluation and management of structural heart disease, thyroid dysfunction, alcohol or drug abuse, or other primary causes of AF is recommended before engaging in sports. Recommending against high-contact or trauma-prone sports for anticoagulated patients.
2020 Canadian Cardiovascular Society Guidelines	Engage in at least 200 minutes per week of moderate-intensity aerobic exercise, along with resistance and flexibility training, with individualized exercise prescriptions based on patient-specific characteristics.

ESC, European Society of Cardiology.

## 7. Evidence Gaps

The relationship between exercise and AF has been extensively studied, yet several evidence gaps persist. A major challenge is the heterogeneity in exercise protocols, including aerobic training, resistance training, and HIIT, making it difficult to establish standardized recommendations. Inconsistencies in study designs, including varying intensity metrics (VO_2_ max percentages, METs, Borg scales), different adherence monitoring methods (self-reports, supervised sessions, wearable devices), and outcome measures further complicated efforts to identify the most effective exercise strategies for AF management [35, 51, 72]. Additionally, most studies have short follow-up durations, limiting the understanding of the long-term effects of exercise on AF recurrence, burden, and cardiovascular outcomes [35, 72]. The optimal exercise intensity and type for reducing AF burden without increasing risk are also not well-defined; while MICT is generally recommended, the role and long-term safety of HIIT require further exploration [7, 72]. Lastly, while there is evidence linking exercise to changes in atrial remodeling, autonomic tone, and inflammation, significant gaps remain in understanding the precise mechanisms and their clinical implications [8].

## 8. Future Direction

Future research on the effects of different exercise modalities and intensities on AF outcomes should address several key areas. Large, multicenter RCTs with long-term follow-up are needed to assess the impact of different exercise modalities on AF outcomes and to establish optimal exercise prescription for AF patients [31, 35, 72]. For example, the ongoing Cologne ExAfib trial is investigating the feasibility and safety of various exercise interventions, including HIIT, MICT, and strength training in patients with paroxysmal AF [30, 31]. Longitudinal studies are equally important to assess the long-term impact of exercise training on AF outcomes, as existing evidence suggests potential benefits in rate control, functional capacity, and QoL. However, further confirmation is needed [73]. Continuous ambulatory monitoring should be integrated into future studies to assess AF burden and exercise effects accurately [24]. Wearable technology and mobile health (mHealth) advances can improve exercise monitoring by providing real-time heart rate and rhythm data, enabling personalized, data-driven exercise recommendations. This approach can minimize recall bias while providing objective quantitative analyses [74]. Future research should also focus on developing personalized exercise programs tailored to maximize benefits, based on individual risk profiles, including age, sex, comorbidities, and baseline fitness levels [1, 34]. Exploration of exercise-related genetic polymorphisms, particularly in the peroxisome proliferator-activated receptor gamma coactivator 1-alpha (PGC-1α), and their impact on AF risk may offer novel insights into patient-specific responses to exercise and further personalize therapeutic strategies [75]. A more in-depth investigation of the underlying mechanisms by which exercise affects AF, such as metabolism, inflammation, atrial stretch, and autonomic modulation, is necessary to identify biomarkers and refine interventions [8, 45].

## 9. Conclusion

Moderate exercise can improve cardiovascular health and QoL in patients with AF while reducing the burden of AF. However, the effects of high-intensity exercise remain uncertain and may vary among individuals. Ongoing research should focus on refining exercise recommendations, aiming to optimize clinical outcomes by balancing the benefits of physical activity with potential risks in AF management.

## Abbreviations

AF, atrial fibrillation; CRF, cardiorespiratory fitness; HR, hazard ratio; OR, odds ratio; QoL, quality of life; RCT, randomized clinical trial; SMD, standardized mean difference.
